# Process service quality evaluation based on Dempster-Shafer theory and support vector machine

**DOI:** 10.1371/journal.pone.0189189

**Published:** 2017-12-08

**Authors:** Feng-Que Pei, Dong-Bo Li, Yi-Fei Tong, Fei He

**Affiliations:** School of Mechanical Engineering, Nanjing University of Science & Technology, Nanjing, Jiangsu, China; Southwest University, CHINA

## Abstract

Human involvement influences traditional service quality evaluations, which triggers an evaluation’s low accuracy, poor reliability and less impressive predictability. This paper proposes a method by employing a support vector machine (SVM) and Dempster-Shafer evidence theory to evaluate the service quality of a production process by handling a high number of input features with a low sampling data set, which is called SVMs-DS. Features that can affect production quality are extracted by a large number of sensors. Preprocessing steps such as feature simplification and normalization are reduced. Based on three individual SVM models, the basic probability assignments (BPAs) are constructed, which can help the evaluation in a qualitative and quantitative way. The process service quality evaluation results are validated by the Dempster rules; the decision threshold to resolve conflicting results is generated from three SVM models. A case study is presented to demonstrate the effectiveness of the SVMs-DS method.

## Introduction

With the proposal of Made in China 2025, intelligent manufacturing has become a new research topic, and manufacturing remains the most vital component of industry. In intelligent manufacturing, the manufacturing activity is considered a type of service. How to assess the service is an urgent problem for manufacturing enterprises. There have been increasingly rapid advances in this field. Many researchers believe that process evaluation of multi-source manufacturing information is a useful measure, which is flourishing although less studied [1].

Some scholars have researched the evaluation of the process. To improve service quality, Zhang Y. [2] proposed a new three-dimensional analysis model of “service attributes, work flows and interactive contacts”. With factor analysis, the system of evaluating the quality of engineering survey (SQES) was tested to demonstrate the evaluation process. A double-layer fuzzy evaluation model was proposed to evaluate engineering service quality by Zong Q. [3]. The fuzziness and randomness of service quality were taken into consideration synthetically in this model. Hrehova S. [4] showed the possibility of creating a predictive model for quality evaluation of the production process with MATLAB by using the basic tools of statistical process control and their graphic representation. Wang L. et al. [5] established a service quality gap model based on the production, management and application of the leaf area index product. A Remote Sensing Technical System of Evaluating Service Quality of Index Products (REI) on leaf area was proposed. There is limited research on service quality evaluation of the adaptive production system [6]. Moreover, most researchers focus on feature weighting for evaluation. Traditional scoring and defective deduction are widely used with intense subjectivity and tendency for quality assessment.

Single feature evaluation is widespread. Some scholars tend to study rough set theory, which combines the similarity and prioritization of a single feature to evaluate production process service quality. Che H. [7] believed that the feature could be extracted in different spatial domains such as the time domain, time frequency domain, and wavelet domain. The information collected was a type of reflection of a device. Chen H. [8] combined brightness, contrast and a variety of other factors as single feature, image distortion factors to evaluate the quality of service. Shalet K. [9] extracted auto-regressive and moving average model features (ARMA) from vibration signals to improve machine performance. However, unreliable, incomplete and indistinguishable datasets can easily lead to the distortion and nonsense of the results. Therefore, the single feature method has been gradually replaced by multi-feature pattern recognition and evaluation [10].

The last few years have seen the development of multi-feature evaluation. Yang G. [11] proposed an effective multi-feature weighted multi-resolution image fusion algorithm. The fusion weights were calculated by averaging gradient feature adaptive weighting. The multi-feature (edge features and average gradient features of a low frequency coefficient and correlation signal intensity ratio of high frequency coefficient) evaluation performed well. Alabi A. [12] used workability and efficiency as the two features to evaluate the performance of the proposed Eigenface algorithm for the recognition of faces and demonstrated better performance of the Eigenface algorithm for the distinct features compared to those with plain features. Audhkhasi K. [13] extracted and demonstrated two features of both long-term and short-term distortions with a two-scale non-intrusive auditory system to evaluate speech quality with good results. Although the accuracy of multi-feature evaluation has been improved [14], there still exist quite a few problems: the complexity and dimension of feature space are relatively high [15], and fusion results, which are simply synthesis, are not precise, real-time and stable evaluation results [16].

Currently, there is increasing interest in using a SVMs-DS approach to further improve the accuracy, efficiency and stability of service quality evaluation especially in the area of fault detection. A fault diagnosis method based on the hierarchical support vector machine (HSVM) and Dempster-Shafer (D-S) theory for analog circuits was designed by Tang J. [17]. The output voltage signals from the test nodes were obtained from analog circuit test points, and faulty feature vectors were extracted from Haar wavelet transform coefficients; then, an HSVM model was built. Conflicting evidence was fused through D-S theory. Zhou C. [18] believed that noise could cause the least squares support vector machine (LS-SVM) to be less effective. More reliable modeling based on robust LS-SVM and D-S theory was proposed. The evidence dataset was developed by a distributed LS-SVM. Conflicting evidence was further fused by the D-S theory. This robust model could represent the original system well even in the presence of different types of random noise. A fault diagnosis method for a wheeled service robot driving system based on multi-principle component analysis (multi-PCA) models was suggested by Yuan X. [19], which compounds SVM and D-S evidence theory. The multi-PCA models are used to extract features from sensor data, and then the data are used in the SVM classifiers. The basic probability assignment (BPA) is determined by the integration of the confidence values.

Based on the above literature review, the existing evaluation models include single feature evaluation, multi-feature weight fuzzy evaluation and multi-feature fusion evaluation. Single feature evaluation cannot fully reflect the production system [20]; objectivity and fairness are weak in multi-feature weight fuzzy evaluation [21]; and multi feature fusion, widely used in fault detection, is another good evaluation method for service quality. Multi-feature fusion cannot make full use of all the feature information. Therefore, in this paper, a new service quality evaluation model based on SVMs-DS is proposed, which can utilize the aggregate information collected. This is particularly needed for a production process such as solar cells, a process where it is not easy to extract production quality features. Section 2 presents the method of SVM, D-S theory and the proposed service quality evaluation model based on SVMs-DS. In Section 3, a case study is submitted to demonstrate the application of the proposed model. Finally, the discussion and conclusion are provided in Sections 4 and 5, respectively.

## Materials and methods

### Support vector machine (SVM)

A support vector machine (SVM) is a statistical supervised machine learning technique, used both for classification and for regression purposes [22],originally proposed by Vapnik and Cortesin 1995 [23]. The original SVM proposal is aimed at both the binary classification problem and the multi-class classification problem [22].

SVM is one of the most successful methods of machine learning, which integrates the maximum separation hyperplane decision boundary, Mercher kernel, convex quadratic function and slack variables. It is used primarily to solve nonlinear classification and regression problems. In recent years, it has gradually been applied for sample analysis, factor selection, information compression, knowledge mining, etc.

#### The basic principle of SVM

The decision boundary hyperplane in SVM classification is calculated by employing a training dataset. This decision boundary is completely defined by the support vectors, a subset of training input vectors, which by themselves lead to the same decision boundary [22]. The sample space is mapped to a high or infinite dimensional feature space (Hilbert space, a generalization of Euclidean space [24]) where linear learning machines can be applied to solve the problem of highly nonlinear classification and regression by using Mercer kernel. The SVM classifier is ready to be used with a different dataset than the one used in the training stage [25].

The nonlinear mathematical model for the SVM based on kernel methods is:

$$maxJ(\alpha )=\max\left(\sum_{i=1}^{l}\alpha_{i}-\frac{1}{2}\sum_{i=1\;j=1}^{l}\alpha_{i}\alpha_{j}y_{i}y_{j}(\varphi (X_{i})∙\varphi (X_{j}))\right)$$

$$s.t.\;\sum_{i=1}^{l}\alpha_{i}y_{i}=0\;(C\geq \alpha_{i}\geq 0;\;i=1,\ldots l)$$ (1)

The number of samples is *l*. *X*_*i*_*ϵR*^*l*^, *y*_*i*_*ϵ*{+1,−1}, *α*_*i*_ is the Lagrange multipliers, and*C* is the penalty factor. *φ*(*X*_*i*_) is the nonlinear transform of *X*_*i*_.

Finally, the optimal hyperplane decision function is obtained:

$$M(X)=sgn(\sum_{\begin{array}{c} \mathrm{Support}\\ \mathrm{vectors} \end{array}}\alpha_{i}^{*}y_{i}(K(X,Y))+b^{*})$$ (2)

where *K*(***X***,***Y***) is the kernel function.Different inner functions in SVM can form different algorithms, and the commonly used kernel functions are as follows:

- Gaussian radial basis function (RBF)
  $$K(X,Y)=exp[-{\left|X-Y\right|}^{2}/\sigma^{2}]$$ (3)
  where σ is a Gaussian parameter.

Each base function center of this classifier corresponds to a support vector; the base function centers with their output weights can be automatically determined by the algorithm. This is the major difference between this RBF classifier and the traditional one.

- Polynomial kernel function

$$K(X,Y)={\left[(X\cdot Y)+1\right]}^{q}$$ 4)

This is a *q*-order polynomial classifier.

- Sigmoid function

K(X,Y)=tanh(v(X∙Y)+c] 5)

Here, SVM forms a multi-layer perception classifier with hidden layers. The number of hidden nodes is automatically determined by the algorithm, which does not have local minimum value trouble.

- Linear kernel function

K(X,Y)=φ(X)∙φ(Y) (6)

In addition to the above kernel functions, there are also other kernel functions such as exponential kernel, Laplacian kernel, multi-quadric kernel, rational quadratic kernel, and so on.

#### The selection of SVM parameters

The penalty factor *C* and parameters of SVM kernel function (such as *g* or *σ* in RBF) are all referred to as SVM parameters. The penalty factor *C* adjusts the confidence interval and the empirical risk ratio for machine learning within a data subspace to improve the learning machine’s extension performance.

Within the data subspace, a small value of *C* indicates a small penalty for the empirical error and a less complex learning machine where the empirical risk can be increased. This is the ‘lack of learning’ case. The opposite of the upper case is recognized as the ‘over learning’ case. There is at least one suitable *C* in each data subspace available to be an optimized SVM classifier. When *C* exceeds a certain value, the complexity of SVM reaches the maximum allowed in the data subspace. In this case, the empirical risk and the generalization ability are almost fixed. However, there is not any uniform method to determine the optimal *C*.

Experiments lead by Chang C. [26] show that as *C* increases, the test accuracy will increase. The number of support vectors will decrease, and the number of support vectors at the boundary will decrease rapidly until there is no support vector at the boundary. Based on experiments [27], *C* has a great impact on training results. The optimal value of *C* depends on a specific condition. In general, for a change of *C*, the larger the amount of data used for training is, the less susceptible the training result. If the training quantity is small, a larger *C* value (allowed by the system) is better. Therefore, it is indicative that if sample quantities are unequal, *C* should be inversely proportional to a sample quantity.*g* is the gamma function set in the kernel function, and its default value is:

$$g=\frac{1}{The\;Numbers\;of\;features}$$ (7)

That is, *g* is the reciprocal of feature quantity, where *g* = 1/2*σ*^2, *σ* is a Gaussian parameter.

### D-S evidence theory

The Dempster-Shafer (D-S) theory is useful for handling uncertainty and imprecision and does not require assigning prior probabilities. D-S evidence theory is a generalization of Bayesian theory. D-S evidence theory can tell ‘uncertain’ and ‘do not know’ without prior probability, which ensures D-S evidence theory’s excellent generalization performance. Based on above characteristics, D-S evidence theory is often used to address multi-source, uncertain information. Based on the recognition framework, denoted by ***Θ*** (elements in ***Θ*** are mutually exclusive), D-S evidence theory is a complete set of all possible answers to a problem.

Definition 1: Let ***Θ*** = {***A***_**1**_,***A***_**2**_,…***A***_***n***_} be the universe; it is the recognition framework. The basic probability assignment (BPA) function *m* is a mapping from $2^{A_{i}}$ to [0, 1], which consists of all possible sub-sets of ***Θ***, including the empty set. ***A***_***i***_ is a subset of the recognition framework ***Θ*** (denoted as *A*_*i*_ ⊆ ***Θ***), and the following is satisfied:

$$\left\{ \begin{array}{c} m(Ø)=0\\ \sum_{A_{i}\subset \Theta }m(A_{i})=1 \end{array}\right.$$ (8)

In this formula, *m*(*A*_*i*_) is called a basic probability assignment (BPA function or mass function) function of *A*_*i*_, and *A*_*i*_ is a given member ofthe power set. The mass *m*(*A*_*i*_) offers all relevant and availableevidence that supports the claim that the actual state belongs to *A*_*i*_.

Definition 2: The Belief function (*Bel*) is a mapping from the set $2^{A_{i}}$ to [0, 1]. A is a subset of the recognition framework ***Θ*** = {***A***_**1**_,***A***_**2**_,…***A***_***n***_} (denoted as *A*_*i*_ ⊆ ***Θ***);the following is satisfied:

Bel(Ø)=m(Ø)=0

$$Bel(\Theta )=\sum_{A_{i}\subset \Theta }m(A_{i})=1$$ (9)

$$Bel(A_{1}\cup \ldots \cup A_{n})\geq \sum_{i}Bel(A_{i})-\sum_{i\neq j}Bel(A_{i}\cap A_{j})+\ldots +{\left(-1\right)}^{n}Bel(A_{1}\cap \ldots \cap A_{n})$$

Bel: $2^{A_{i}}\to$ [0, 1] is called a reliability function.

When *m*(*θ*) > 0, ***Θ*** is the focal element of the function *Bel*: $2^{A_{i}}\to$ [0, 1] and the kernel of the recognition function ***Θ***.

According to D-S theory, the quantity $Pl(A)=1-Bel(\bar{A})$, where $\bar{A}$ is the negation of *A*, is called the plausibility of *A*. Generally, we have [28]

Bel(A)≤P(A)≤Pl(A)

where *P*(*A*) is the probability of *A*.

From this inequality, a body of evidence about some set of hypotheses may provide a set of compatible probabilities bounded by a belief function *Bel*(*A*) and a plausibility function *Pl*(*A*) [29], even if the system information is insufficient or the system is disturbed by various kinds of stochastic noise. This means that the boundaries of the real system output can be calculated even if there is either Gaussian noise or other kinds of stochastic uncertainty in the data. In this way, the true system information can be extracted from the data in a belief function using D-S theory.

#### Dempster rule

In D-S theory, different independent sets of masses can be aggregated using the Dempster rule of combination. A commonly shared belief between multiple sources is extracted, and all conflicting (non-shared) beliefs are ignored [30–31]. Generally, let *Bel*_1_ and *Bel*_2_ be two belief functions on the same framework *U*; *m*_1_ and *m*_2_ are their basic probability assignments (BPAs), respectively, and their focal elements are *A*_1_…*A*_*k*_ and *B*_1_…*B*_*k*_,

$$k=\sum_{A_{i}⋂B_{j}=\emptyset }m_{1}(A_{i})m_{2}(B_{j})$$ (10)

Then:

$$m(C)=\left\{ \begin{array}{cc} \frac{\sum_{A_{i}⋂B_{i}=C}m_{1}(A_{i})m_{2}(B_{j})}{k}, & \forall C\subset UC\neq \emptyset \\ 0, & C=\emptyset \end{array}\right.$$ (11)

*k* is the conflict factor, which reflects the conflict degree of the evidence.

#### Decision-making rule

If *A*_1_,*A*_2_ ⊂ *U* and they satisfy:

$$m(A_{1})=max\left\{m(A_{i}),A_{i}\subset U\right\}$$

$$m(A_{2})=max\{m(A_{i}),A_{i}\subset U,A_{i}\neq A_{1}\}$$ (12)

If there are:

$$\left\{ \begin{array}{c} m(A_{1})-m(A_{2})>\varepsilon_{1}\\ m(\Theta )<\varepsilon_{2}\\ m(A_{1})>\varepsilon_{3}>m(\Theta ) \end{array}\right.$$ (13)

Then, *A*_1_ is the decision result, where *ε*_1_, *ε*_2_, *ε*_3_ are preset thresholds, and ***Θ*** is an uncertain set.

D-S evidence theory has several defects: it cannot address serious conflicts and complete conflicts; it is difficult for this theory to identify the fuzzy degree of synthetic evidence; the more elements in the subset there are, the fuzzier the subset; and so on. There is an army of improved algorithms proposed by domestic and overseas scholars, such as the Yager algorithm, improved Yager algorithm, weighted evidence combination method, weighted assignment conflict method, weighted absorption method based on confidence, etc. [32].

### The proposed algorithm of SVMs-DS

**Fig 1:**
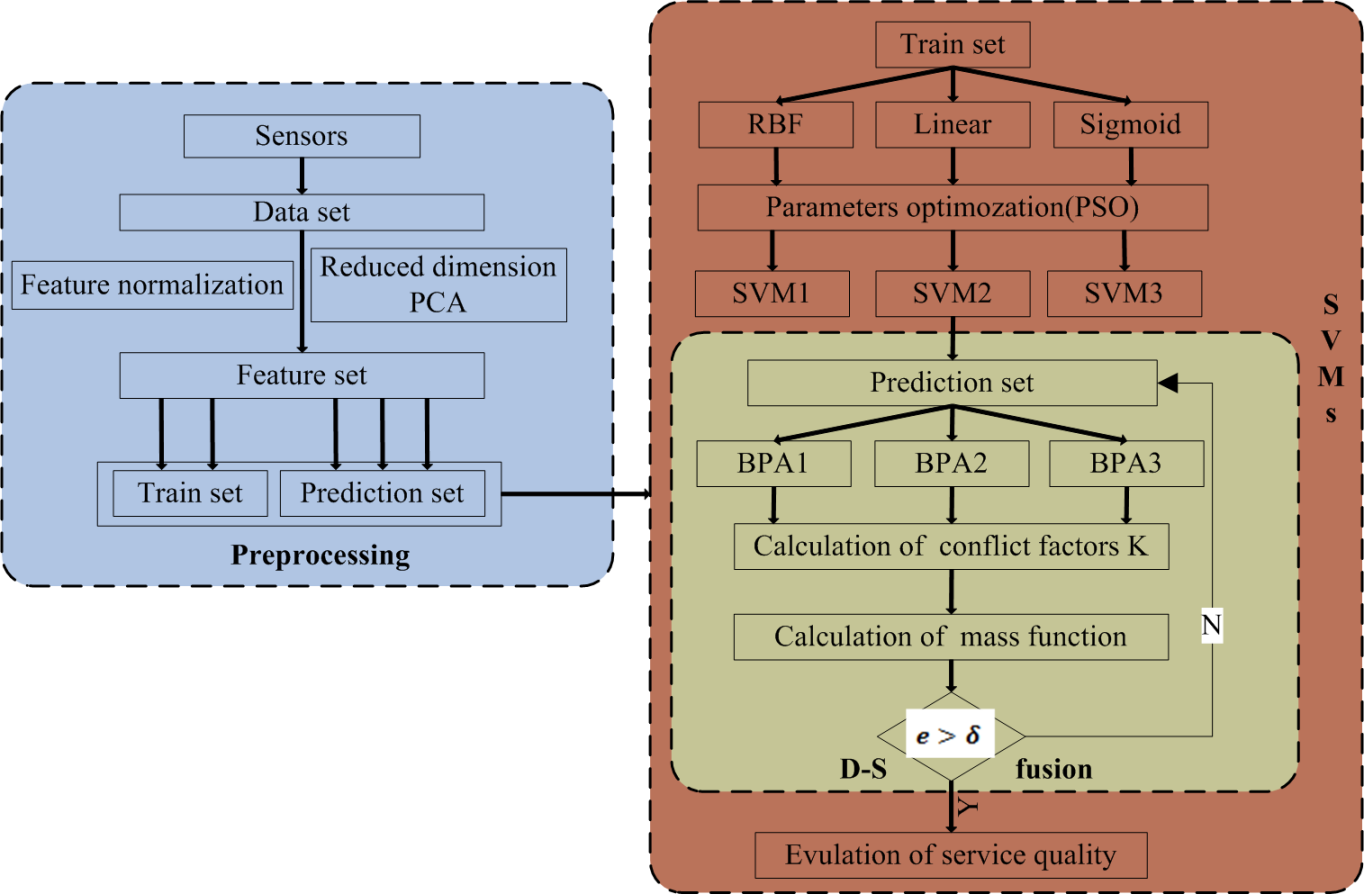
The proposed algorithm of SVMs-DS.

The proposed quality evaluation requires three SVM structures with individual kernel functions for the evaluation; each SVM is independent from the others. Therefore, the different SVM may provide contradictory evaluation results. The proposed method employs the D-S theory to combine the contradictory accuracy (the BPAs) in the testing phase; the conflicting results generated in the testing phase can be refined. The three kernel-function-SVM-structures are one of the innovations of this paper. The proposed algorithm of SVMs-DS is shown in Fig 1.

Input: the feature set.

Output: the fusion result of service quality evaluation.

STEP1: The data set is constructed from the information collected from sensors.

STEP2: The feature set is generated after the normalization and dimensionality reduction.

STEP3: Trained by certain kernel function. The results of preprocessing are randomly selected as the training set, and the remaining are the prediction set. It is necessary to set the parameters or to optimize the parameters in the SVM before training. The feature space can be classified into multiple hyperplane decision boundaries of different spaces after the SVM training. There are 3 different kernel functions (RBF, Linear, and Sigmoid) for training. The results are labeled SVM1/SVM2/SVM3.

STEP4: Test the prediction set by the SVM1/SVM2/SVM3 and obtain the basic probability assignments (BPAs). Voting mechanisms are put in place for BPAs.

STEP5: Fuse the BPAs by D-S theory. The BPAs will serve as the independent evidence of D-S theory for input. After calculation, the conflict factors *K* and mass function, the fusion result of service quality evaluation, will be assessed by the decision threshold. The service quality evaluation is issued.

## Case study

### Preprocessing of the feature set

#### The data set (STEP1)

**Fig 2:**
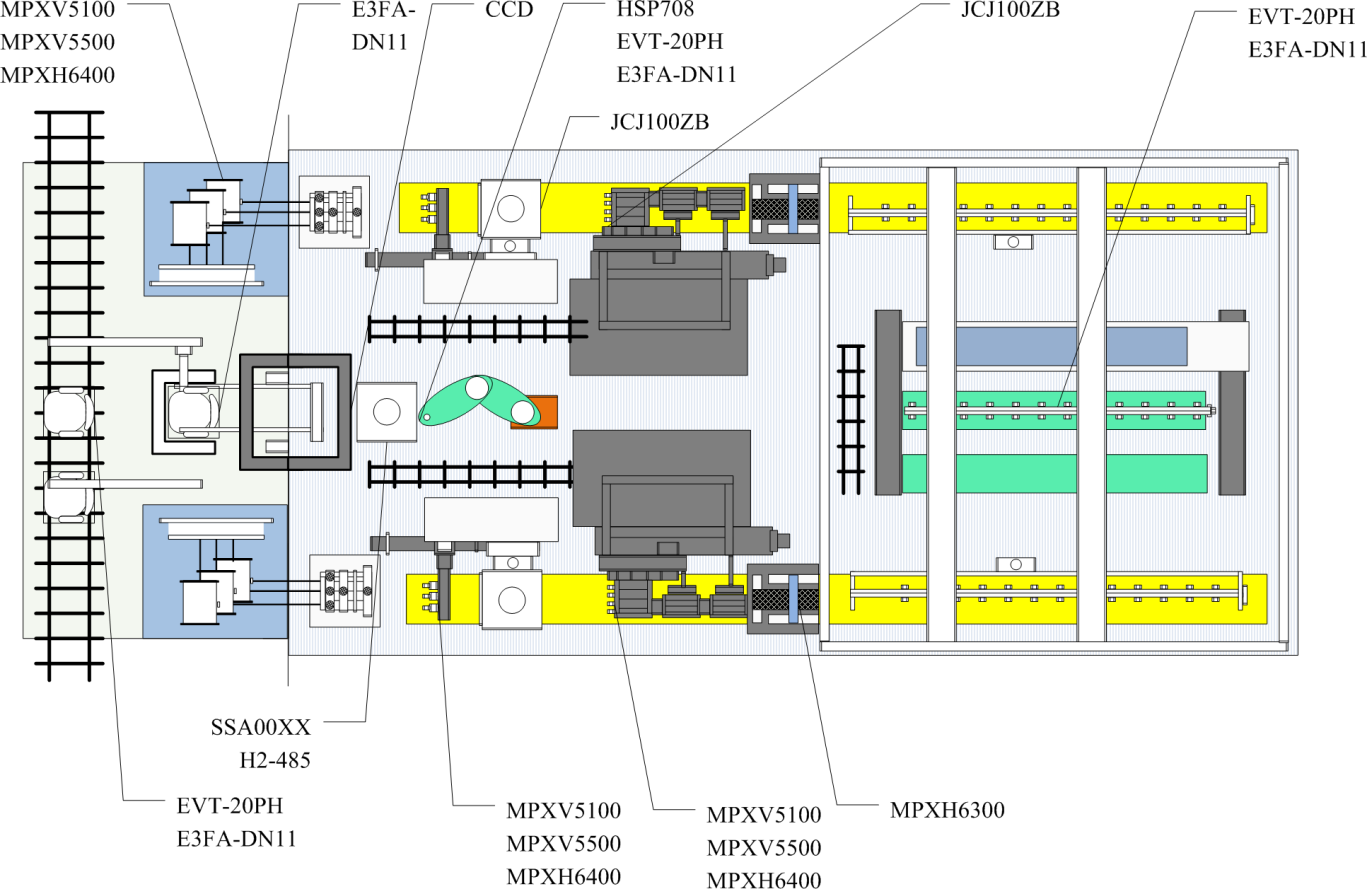
Key sensors in the production line.

The original data are collected by sensors and other measurements. Therefore, the first step is to install a large number of sensors. Some key sensors are shown in Fig 2.

**Fig 3:**
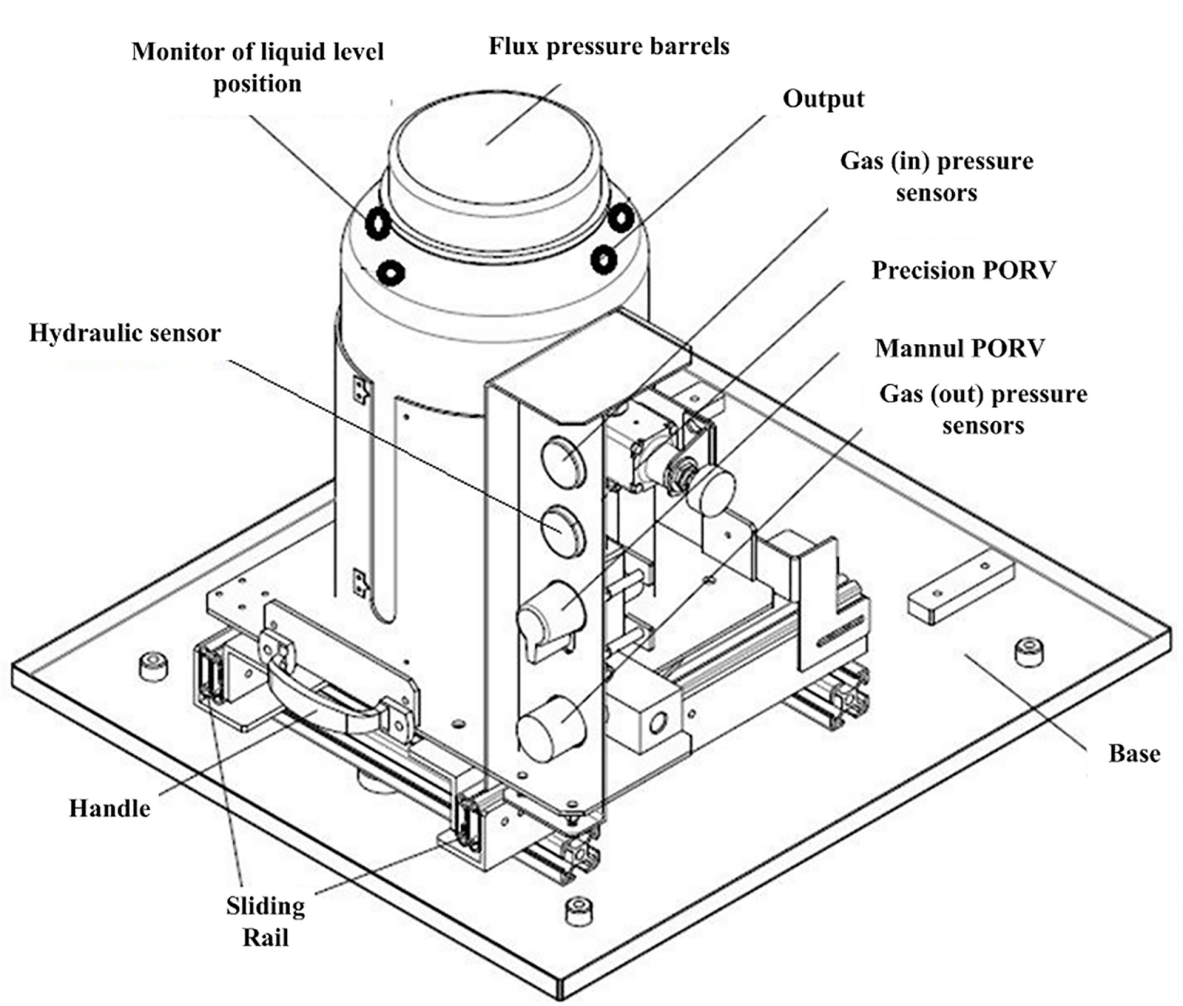
The monitor of liquid level position.

In Fig 2, there are some key sensors in the production line. MPXH6300/MPXV5100/MPXV5500/MPXH6400 are the pressure sensors used to estimate the clamp force/cutting force of air. Their differences are ranges and accuracies. EVT-20PH and E3FA-DN11 are utilized to count the number of solar cells. SSA00XXH2-485 determines the deflection angles, and JCJ100ZB measures temperature. The CCD is edge detection. The gas/hydraulic sensors are all used to monitor the liquid level position indicated in Fig 3.

The solar string welder production process can be divided into several systems: feeding system, loading system, CCD vision system, manipulator transfer system, release system, pressure belt system, cutting mechanism system, welding platform systems, photovoltaic welding systems, film system, lateral transfer system, flip mechanism and so on. In total, there are 309 features collected as shown in Table 1.

**Table 1:** The features of the production process.

System	Features and Numbers
Production preparation process	Belt/Flux/Air pressure battery separator air blowing/servo reset/reset/fault detection, etc. 43 items in total.
Feeding system	The main grid direction of cell/the number of feed/step forward or backward/step specifications, etc. 13 items in total.
Loading system	Status of suction fan/Suction position of cell discharge area/separation air knife/cylinder waiting position/grab position, etc. 18 items in total.
CCD vision system	Number of NG pieces/battery plate specifications/cell edge detection/silk screen detection/corner detection/grid line detection/calibration of the standard features, etc. 66 items in total.
Manipulator transfer system	Reset button/ROB connection status/fetch to CCD platform/battery box to CCD/battery box to the adjust platform/the adjust platform to the CCD camera features, etc. 20 items in total.
Release system	Continuous welding traction/tension tightened state, etc. 8 items in total.
Pressure belt system	Fault status/welding band bending/bending cylinder, etc. 7 items in total.
Cutting mechanism system	Tape length/tail-tail empty state and length/number of cells/cut-and-hold state, etc. 18 items in total.
Welding platform system	Insulation state and insulation temperature/cooling state and cooling temperature, etc. 15 items in total.
Photovoltaic welding systems	Real-time production/welding temperature/traction jaw/welding time/conveyor speed, etc. 25 items in total.
Film system	Film material selection/film to tighten/film transfer mechanism state/floor temperature conditions, etc. 12 items in total.
Lateral transfer system	Lateral movement state/full number of NG box/finished box full inspection, etc. 11 items in total.
Flip mechanism	Transport state/adsorption mechanism state/adsorption flip state, etc. 9 items in total.
To be classified	Discharge OK/NG inspection/cumulative capacity/welding frequency/number of welding/the number of NG/machine speed/welding light power, etc. 44 items in total.

#### Feature set (STEP2)

Eliminate the singular features, such as major equipment failure and solar string welder switches, for the above 309 features by data screening based on rules. After feature elimination, a feature set containing 86 values is constructed as shown in Table 2.

**Table 2:** The standard value of features.

Features	Standard	Features	Standard	Features	Standard
Welding temperature	Number	Film transfer mechanism	0/1/2	Numbers of every battery string	1–30
The count of cells in one piece	1–12	Gas welding pieces	0–100	Number of site battery strings	Number
Pressure of Flux spray(MPa)	0.05–0.09	Checking box weight of 90 degrees flips	270–330	The delay of putting down the pieces	100–3000
Full strings number of NG box	1–30	Last string offset length	0–10	Belt correction(mm)	0–10
"Feed Forward" button count	1-n	Pieces produced	Number	Material selection	0/1/2/3/4
The length of welding time	1500–300	Extension of welding time	0–200	1–4#Preheating temperature	0–200
NG alarm limit	15/25	Edge defects	1/2/3/4	180 degrees flip	0131/0132
Screen offset	100–250	The number under one piece	Number	Position of welding device	0/1/2/3
90 degrees flip	0/1/3/4/5/6/7	Air pressure(MPa)	0.55–0.70	Cell spacing	0–6
Screen offset level	0005–0015	Power of lamp (%)	0/100	Power of welding light box(%)	0–100
Checking box height of 90 degrees flips	270–330	Acceleration time of conveyor belt	100–2000	Deceleration time of conveyor belt	100–200
—	—	—	—	—	—

Principal component analysis (PCA) is used to lower the dimension of the86 features to simplify the calculation. The main principles of PCA are as follows:

$$\left\{ \begin{array}{c} F_{1}=a_{11}x_{1}+a_{12}x_{2}+\ldots +a_{1p}x_{p}\\ F_{2}=a_{21}x_{1}+a_{22}x_{2}+\ldots +a_{2p}x_{p}\\ \ldots \\ F_{q}=a_{q1}x_{1}+a_{q2}x_{2}+\ldots +a_{qp}x_{p} \end{array},\;q\leq p\in N^{+}\right.$$ (14)

*x*_*i*_ denotes the original eigenvector, *F*_*i*_ denotes the eigenvector analyzed by PCA, and *a*_*ij*_ is a dimension reduction matrix. While 86 features are dimensionally reduced to 57, feature vectors can be normalized into [0, 1].

$$x=\frac{x^{\prime }-x_{min}^{\prime }}{x_{max}^{\prime }-x_{min}^{\prime }}$$ (15)

*x*′ stands for a single vector value, while $x_{min}^{\prime }$ is its minimum value, and $x_{max}^{\prime }$ is its maximum value. *x* is the normalized result of *x*′. If ${x^{\prime }=x}_{min}^{\prime }$, then *x* = 0; if ${x^{\prime }=x}_{max}^{\prime }$, then x = 1. Results are shown in Table 3.

**Table 3:** The normalization of all the samples.

	Feature1	Feature2	…	Feature 55	Feature 56	Feature 57
Sample1	0.409396	0.222222	…	0.73	0.975	0.316583
Sample 2	0.278523	0.703704	…	0.58	0.45	0.924623
Sample 3	0.85906	0.398148	…	0.91	0.875	0.035176
Sample 4	0.177852	0.074074	…	0.64	0.725	0.723618
Sample 5	0.325503	0.833333	…	0.97	0.275	0.884422
Sample 6	0.97651	0.259259	…	0.73	0.425	0.417085
…	…	…	…	…	…	…
Sample 192	0.137584	0.111111	…	0.88	0.475	0.050251
Sample 193	0.177852	0.157407	…	0.4	0.725	0.844221
Sample 194	0.83557	0.87963	…	0.07	0.45	0.211055
Sample 195	0.483221	0.722222	…	0.28	1	1
Sample 196	0.902685	0.861111	…	0.19	0.2	0.984925

### Train the set by the RBF/linear/Sigmoid function(STEP3)

Since there is a total of 196 samples for the process service quality evaluation,150 samples are randomly selected as the training set to synthesize the model, and the remaining 46 samples form the prediction set to test the model. The data can be found in S1 Dataset.

Set parameter *C* = 1.828 and *g* = 0.1 (calculated by LibSVM, *c*_1_ = 1.5 and *c*_2_ = 1.7 are the optimized parameters deduced by 100 iterations;software available at http://www.csie.ntu.edu.tw/~cjlin/libsvm).

First, non-linear dataset *p*{*label*: *feature set*} maps the space sample into a high/infinite dimension feature space (Hibert space), which transforms the non-linear problem in the original sample space into a linear problem in the feature space. Then, hyperplane decision boundary models are built by training those 150 samples using SVM based on RBF, Linear and Sigmoid kernel functions.

### Test the prediction set (STEP4)

The remaining 46 samples are the prediction set.

Test the hyperplane decision boundary models built above by using the test set. The test result, which used SVM based on RBF, shows that 3 samples have calculation inconformity to the evaluation given by the enterprise of ATW. SVM based on Linear kernel have2 and SVM based on Sigmoid kernel have 4. Table 4 shows the test results.

**Table 4:** The results after the SVMs in three different kernel functions.

ATW	RBF SVM1	Linear SVM2	Sigmoid SVM3	Feature1	…	Feature 57
5	5	4	5	2.36	…	52
2	2	2	3	0.67	…	187
2	1	2	1	2.29	…	198
2	2	2	3	0.57	…	5
3	3	2	3	0.8	…	195
3	3	3	5	0.63	…	137
2	3	2	2	0.29	…	158
3	5	3	3	0.64	…	169

As is shown in Table 4, the label of the first sample is 5, which means that the daily production service is evaluated as level 5 by an engineer. There are 5 different levels in the ATW to show different service quality. Hyperplane decision boundary deduced by SVM based on Linear kernel (denoted as SVM2) is evaluated as level 4. Hyperplane decision boundaries deduced by SVM based on RBF and Sigmoid kernel are evaluated as level 5. Among them, the BPA results of all tests are shown in Table 5.

**Table 5:** The BPAs of the contradictory results.

Kernel	ATW	Predict	Level 1	Level2	Level3	Level4	Level5
RBF	5	5	0.009204	0.008681	0.06878	0.626556	0.286779
	2	2	0.023804	0.534545	0.309293	0.079959	0.052399
	2	1	0.478178	0.462232	0.037892	0.015048	0.00665
	2	2	0.006582	0.6427	0.3004	0.037672	0.012621
	3	3	0.05187	0.322311	0.29788	0.255951	0.071988
	3	3	0.017846	0.037589	0.541809	0.089278	0.313479
	2	3	0.010682	0.4041	0.4766	0.086518	0.0221
	3	5	0.018971	0.046442	0.423176	0.075565	0.425847
Linear	5	4	0.006913	0.045982	0.213967	0.247951	0.485187
	2	2	0.115679	0.411837	0.292835	0.094925	0.084724
	2	2	0.110726	0.655221	0.120995	0.062552	0.050506
	2	2	0.035896	0.6156	0.2706	0.023049	0.054846
	3	2	0.073522	0.187599	0.319974	0.260061	0.158843
	3	3	0.042226	0.073972	0.569167	0.085034	0.229602
	2	2	0.030435	0.615	0.3176	0.019965	0.01703
	3	3	0.043486	0.049395	0.619525	0.03028	0.257313
Sigmoid	5	5	0.010895	0.0121	0.051024	0.25966	0.666321
	2	3	0.024652	0.394896	0.446953	0.090885	0.042615
	2	1	0.491324	0.462232	0.026476	0.013798	0.00617
	2	3	0.004927	0.4655	0.4897	0.028835	0.011029
	3	3	0.029397	0.095971	0.467016	0.34441	0.063206
	3	5	0.012669	0.033449	0.373827	0.003689	0.576366
	2	2	0.082888	0.5326	0.2769	0.074016	0.033545
	3	3	0.00914	0.017691	0.700537	0.021671	0.250961

### Recognition framework and the results (STEP5)

BPAs by different kernel functions in each sample do not depend on each other and can serve as inputs to D-S evidence theory [33]. Before fusion, a recognition framework should be built: the BPA of the ATW’s evaluation level is one subset of the recognition framework (denoted as ATWBPA, m(A_1_)); the BPA of the prediction level is another subset of the recognition framework (denoted as PPEBPA, m(A_2_)); and the sum of the other levels’ BPAs is the last subset, uncertain information, *m(****Θ****)*.This study uses the first sample in Table 4 as an example. The first sample’s ATW level/RBF SVM/Sigmoid SVM is level 5, and Linear SVM is level 4. They clash with each other. The BPA of level 5 of each kernel function SVM is the ATWBPA (*m*(*A*_1_)); the BPA of level 4 is the PREBPA (*m*(*A*_2_)), and the *m*(***Θ***),which is recognized as uncertain information, is the sum of the other three levels, as shown in Table 6.

**Table 6:** The recognition framework of the first sample.

Kernel	ATW level	Fusion level	ATWBPA*m*(*A*_1_)	PREBPA*m*(*A*_2_)	*m*(*Θ*)
**RBF**	5	5	0.2868	0.6266	0.0867
**Linear**	5	4	0.4852	0.2480	0.2669
**Sigmoid**	5	5	0.6663	0.2597	0.0740

According to the Dempster rule, the RBF data/Linear data/Sigmoid data will take two steps to obtain the final decision. The first step is to fuse the RBF data and Linear data and calculate the first fusion data (intermediate data). The second step is to fuse the first fusion data with the Sigmoid data.

The conflict factor *k* of the first step is calculated by Eq (10):

$$\begin{array}{l} k=\sum_{A_{i}⋂B_{j}=\emptyset }m_{1}(A_{i})m_{2}(B_{j})\\ \;={ATWBPAm(A_{1})}_{RBF}\times {ATWBPAm(A_{1})}_{Linear}+{PREBPAm(A_{2})}_{RBF}\\ \;\times {PREBPAm(A_{2})}_{Linear}+{m(\Theta )}_{RBF}\times {m(\Theta )}_{Linear}=0.3177 \end{array}$$

The BPA of the intermediate data is calculated by Eq (11):

$$\begin{array}{l} m\left(\mathrm{ATWBPA}m{\left(A_{1}\right)}_{\mathrm{RBF}|\mathrm{Linear}}\right)=\frac{\mathrm{ATWBPA}m{\left(A_{1}\right)}_{\mathrm{RBF}}\times \mathrm{ATWBPA}m{\left(A_{1}\right)}_{\mathrm{Linear}}}{k}=0.4380\\ m\left(\mathrm{PREBPA}m{\left(A_{2}\right)}_{\mathrm{RBF}|\mathrm{Linear}}\right)=\frac{\mathrm{PREBPA}m{\left(A_{2}\right)}_{\mathrm{RBF}}\times \mathrm{PREBPA}m{\left(A_{2}\right)}_{\mathrm{Linear}}}{k}=0.4892\\ m\left(m{\left(\Theta \right)}_{\mathrm{RBF}|\mathrm{Linear}}\right)=\frac{m{\left(\Theta \right)}_{\mathrm{RBF}}\times m{\left(\Theta \right)}_{\mathrm{Linear}}}{k}=0.0728 \end{array}$$

*m*(PREBPA*m*(*A*_2_)_RBF|Linear_) > *m*(ATWBPA*m*(*A*_1_)_RBF|Linear_); the first step fusion level is PREBPA (*m*(*A*_2_)) or level 4. Fusion results are shown in Table 7.

**Table 7:** The fusion results of the first step.

Kernel	ATW level	Fusion level	ATWBPA*m*(*A*_1_)	PREBPA*m*(*A*_2_)	*m*(*Θ*)
**RBF**	5	5	0.2868	0.6266	0.0867
**Linear**	5	4	0.4852	0.2480	0.2669
**First-Fusion**	5	4	0.4380	0.4892	0.0728

The second step of the fusion uses the same method as the first step; the fusion results are shown in Table 8.

**Table 8:** The fusion results of the second step.

Kernel	ATW level	Fusion level	ATWBPA*m*(*A*_1_)	PREBPA*m*(*A*_2_)	*m*(*Θ*)
**First-Fusion**	5	4	0.4380	0.4892	0.0728
**Sigmoid**	5	5	0.6663	0.2597	0.0740
**Second-Fusion**	5	5	0.6880	0.2993	0.0127

All the contradictory results in Table 4 are fused, and the fusion results are shown in Table 9.

**Table 9:** The fusion results of the contradictory results.

ATW level	Fusion level	ATWBPA*m*(*A*_1_)	PREBPA*m*(*A*_2_)	*m*(*Θ*)
5	5	0.6880	0.2993	0.0127
2	2	0.6453	0.3005	0.0541
2	2	0.8400	0.1561	0.0039
2	2	0.8212	0.1775	0.0013
3	Uncertain	0.3371	0.0439	0.6190
3	3	0.8624	0.1306	0.0070
2	2	0.7529	0.2384	0.0087
3	3	0.8663	0.1297	0.0040

As shown in Table 9, using the first sample as an example, we obtain the following conclusions.

1) The fusion level is 5, which is equal to the ATW level.

2) In this paper, according to statistics data, the thresholds are set as *ε*_1_ = 0.34; *ε*_2_ = 0.06; *ε*_3_ = 0.6.

*ε*_1_ stands for the difference between *m*(*A*_1_) and *m*(*A*_2_). The minimum *ε*_1_ is one-third; the larger the difference between the two contradictory results is, the higher the credibility.

*ε*_2_ stands for the *m*(*Θ*). It is the proportion of uncertain information. The lower the *m*(*Θ*) is, the better the fusions.

*ε*_3_ stands for the difference between *m*(*A*_1_) and *m*(*Θ*); and the *ε*_3_ sets the minimum *m*(*A*_1_) and the maximum *m*(*Θ*).

The mass function of the ATW (*m*(*A*_1_)) is 0.6880, and the predicted result (*m*(*A*_2_)) is 0.2993, which satisfies the conditions *m*(*A*_1_) − *m*(*A*_2_) > *ε*_1_, *m*(*Θ*) < *ε*_2_, *m*(*A*_1_) > *ε*_3_ > *m*(*Θ*).

Based on the above conclusions, the model is supported by the fusion result. In Table 10, 7/8 samples passed the threshold test.

**Table 10:** Parameters of fusion.

	Iterations	Total number of support vectors	Prediction accuracy
RBF	100	87	93.4783
Linear	149	90	95.6522
Sigmoid	84	117	91.3043
D-S Fusion	-	-	97.8261

## Discussion

The proposed process service quality evaluation method employs the D-S theory to combine the BPAs of each SVM. The prediction results can be refined, and the prediction accuracy increases as shown in Table 10.The total numbers of support vectors deduced from SVM based on RBF, Linear and Sigmoid data are 87, 90 and 117, respectively, without "over learning”. Compared with the prediction accuracy of the RBF (93.4%)/Linear (95.6%)/Sigmoid (91.3%) data, the prediction accuracy of the proposed D-S fusion (97.8%) is higher because of using all features and fusing the three different kernel functions to evaluate the quality of service.

The advantages of the proposed method are:

- Compared with the traditional SVM-DS, the application domain of this paper is new pattern recognition(process service quality evaluation) instead of fault diagnosis. The proposed evaluation method utilizes three different kernel functions to fuse;
- Compared with the single feature evaluation, the proposed method has higher accuracy, smaller fluctuation and stronger stability;
- Compared with the fuzzy weight analysis method, the proposed method is more objectivein machine learning; and
- Compared with the multi-feature evaluation, the proposed approach makes full use of all the features.

## Conclusions

This paper proposes a SVMs-DS method to evaluate service quality. The BPAs are put into the D-S method as independent evidence. Then, the Dempster rule and thresholds are introduced to the algorithm. The SVMs-DS is found to be superior and more accurate for process service quality evaluation.

This method combines three SVMs in parallel for service quality evaluation based on kernel functions. Take each SVM as independent evidence; the evaluation results can be obtained by combining the three individual SVM results and making the ultimate decision by employing D-S evidence theory. The experimental results show that SVM-DS elevates the accuracy and stability of the service quality evaluation, which can obtain 97% prediction accuracy.

Although SVMs-DS can obtain test success of 97.8% by considering suitable sigma and penalty factors, the machine dataset is the only information resource. As a system, the information of operators such as muscle fatigue and brain fatigue should also be considered when evaluating service quality. The collection, extraction and fusion of these physiological data based on neural industrial engineering represent an important direction to devote future research efforts.

## Supporting information

S1 Dataset The dataset of the process service quality evaluation. (MAT)
